# Splicing Regulation: A Molecular Device to Enhance Cancer Cell Adaptation

**DOI:** 10.1155/2015/543067

**Published:** 2015-07-26

**Authors:** Vittoria Pagliarini, Chiara Naro, Claudio Sette

**Affiliations:** ^1^Department of Biomedicine and Prevention, University of Rome Tor Vergata, 00133 Rome, Italy; ^2^Laboratory of Neuroembryology, Fondazione Santa Lucia, 00143 Rome, Italy

## Abstract

Alternative splicing (AS) represents a major resource for eukaryotic cells to expand the coding potential of their genomes and to finely regulate gene expression in response to both intra- and extracellular cues. Cancer cells exploit the flexible nature of the mechanisms controlling AS in order to increase the functional diversity of their proteome. By altering the balance of splice isoforms encoded by human genes or by promoting the expression of aberrant oncogenic splice variants, cancer cells enhance their ability to adapt to the adverse growth conditions of the tumoral microenvironment. Herein, we will review the most relevant cancer-related splicing events and the underlying regulatory mechanisms allowing tumour cells to rapidly adapt to the harsh conditions they may face during the occurrence and development of cancer.

## 1. Introduction

The transcription units of most eukaryotic genes are characterized by exons, short regions of approximately 200 bp containing untranslated and coding sequences, interspersed between large noncoding introns (generally ≥ 1000 bp) [1]. Removal of intronic sequences and joining of exons is one of the key events in the multistep process ensuring maturation of pre-mRNAs into mRNAs [2]. This process, called splicing, is carried out by the spliceosome, a complex macromolecular machinery composed of five small nuclear ribonucleoprotein particles (U1, U2, U4, U5, and U6 snRNP) and a large number of auxiliary proteins [3]. Alternatively, a small proportion of introns (≈1%) are processed by the minor spliceosome comprising U11, U12, U4atac/U6atac, and U5 snRNPs [4]. The main spliceosome mediates the recognition of short consensus sequences defining the 5′ (GU) and 3′ (AG) splice sites (ss) and catalyses the two transesterification reactions necessary for the junction of exons and removal of introns [5]. Beside the conserved dinucleotide sequence that marks the ss, exon-intron junctions are not characterized by a stringent consensus and their short and degenerate nature is not sufficient to ensure perfect recognition by the spliceosome. Thus, additional factors are required to assist the spliceosome in its critical and essential function.

The activity of the spliceosome is regulated by both* cis*-acting sequences on the pre-mRNA and transacting factors, which may enhance or inhibit both recognition of the ss and splicing catalysis [5]. The* cis*-acting regulatory elements are classified according to their location and activity into exonic and intronic splicing enhancers (ESEs and ISEs, resp.) or silencers (ESSs and ISSs, resp.) [6]. These sequence elements are recognized by transacting RNA binding proteins (RBPs), which in turn promote or inhibit spliceosome assembly and activity. Two main classes of RBPs that regulate splicing by binding to these* cis*-acting regulatory elements are the Ser/Arg rich (SR) proteins, which mainly exert a positive regulation on the spliceosome, and the heterogeneous nuclear ribonucleoproteins (hnRNPs), which often act antagonistically and inhibit splicing [7].

Chromatin signatures on the template DNA also participate in splicing regulation. Higher levels of nucleosome occupancy and specific histone modifications, such as trimethylation of H3K36, were found to be enriched in exons [8]. These observations suggest that epigenetic marks may facilitate exon recognition during splicing, perhaps by slowing down RNA polymerase II (RNAPII) in proximity of exons. Indeed, splicing largely occurs cotranscriptionally when the nascent pre-mRNA is still bound to the DNA template [9] and is likely affected by the elongation rate of the polymerase [10]. In addition, some splicing factors (SFs) interact with chromatin-binding proteins (i.e., MRG15, Gcn5, CHD1, and HP1*α*) and are recruited to histone marks enriched nearby exons, thereby modulating their selection [10, 11].

An additional layer of complexity to the splicing process is provided by the presence of exons characterized by even weaker elements defining exon-intron boundaries. Although this feature makes these exons weaker, it also represents a flexible resource for the gene as it allows their variable inclusion into mature transcripts through the alternative splicing (AS) process. Indeed, through differential assortment of weak or variable exons, a gene can yield multiple mRNA splice variants, potentially encoding proteins with different or even opposite function and/or displaying different patterns of spatial/temporal expression [6, 12].

The advent of high-throughput sequencing technologies has revealed the unexpected pervasive nature of AS. It is now clear that the vast majority of higher eukaryotic genes undergo AS [13, 14]. In some cases, the combinatorial nature of AS allows a single gene to encode for up to thousands of mRNA variants. This extreme flexibility of the splicing process contributes to the great expansion of the coding potential and plasticity of the genome [15, 16]. In support of this notion, eukaryotic cells have been documented to promptly modulate their splicing program in response to different intra- and extracellular cues [17], thus making AS a key tool to fine-tune gene expression. AS plays a pivotal role in controlling core cellular processes, such as proliferation, metabolism, and apoptosis, and fundamental physiological decisions, such as maintenance of pluripotent state or induction of a specific differentiation lineage [18]. Nevertheless, although AS represents a key tool to control gene expression in higher organisms, the extreme flexibility and multilayer nature of its regulation render it error prone and susceptible to alterations that threatens the maintenance of cellular homeostasis. As a proof of this concept, aberrant regulation of AS contributes to the onset or progression of several human diseases, including cancer [19, 20].

In the last decade, high-throughput analyses of transcriptomes have highlighted widespread alterations of AS patterns in human cancer [21, 22]. When identified, the causes of these alterations were attributed to almost all the regulatory steps controlling AS [23–25]. Mutations in the* cis*-acting splicing regulatory elements, altered expression of SFs, and aberrant regulation of proteins and signalling pathway regulating their activity have all been documented in cancer cells and identified as factors promoting oncogenic splice variants and contributing to neoplastic transformation or later stages of carcinogenesis [23, 24]. Thus, cancer cells can rapidly adapt to stimuli received from both extracellular and intracellular cues by finely regulating AS in order to shape gene expression. Herein, we will discuss examples of how AS contributes to the enhanced adaptation capability of cancer cells towards the adverse conditions occurring during the tumorigenic process or triggered by therapeutic intervention.

## 2. Functional Impact of Splicing in Cancer Adaptation

**Figure 1:**
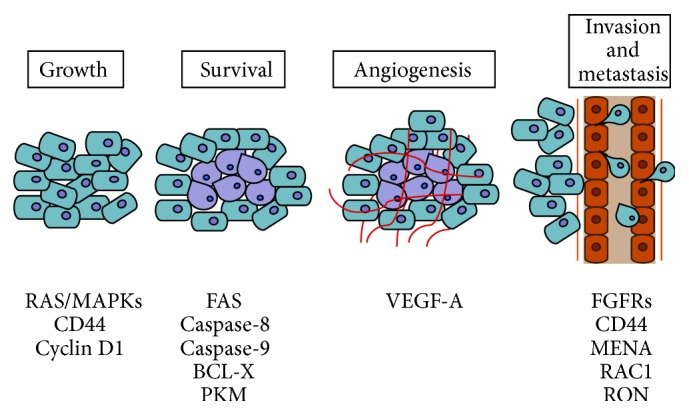
AS events that contribute to the adaptive response of cancer cells during tumorigenesis. Most relevant cancer-related genes undergoing AS misregulation are shown below the key events of tumoral transformation.

**Figure 2:**
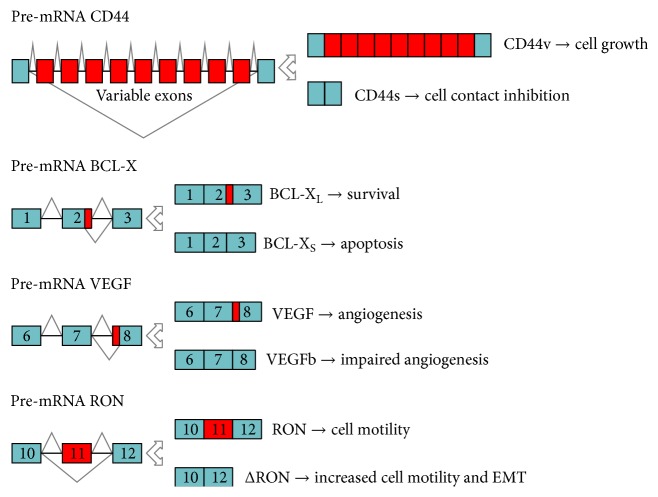
AS events that characterize specific phases of tumour occurrence and development. Green boxes and red boxes indicate constitutive and variable exons, respectively. Black lines indicate the intron sequences. The splice variants produced and their cellular functions are illustrated to the right of the gene schematic representation.

Cancer is a complex disease associated with a variety of genetic and epigenetic aberrations. As illustrated by Hanahan and Weinberg [26], during carcinogenesis cells acquire ten common traits: sustained proliferative signalling, resistance to death, evasion from growth suppressors, ability to invade normal tissues and metastasize, replicative immortality, induction of angiogenesis, genetic diversity generated by genome instability, inflammation, reprogramming of energy metabolism, and escape from immune destruction. A number of studies have now documented that aberrant regulation of AS in cancer cells contributes to many of these traits by allowing the production of oncogenic splice variants from multiple genes (Figure 1). Specific splice variant signatures are strongly associated with particular types of cancers, representing valuable diagnostic and prognostic markers [27, 28]. Although their functional/mechanistic roles are still largely uncharacterised, these splice variants likely contribute to the acquisition of therapeutic resistance and to the increased adaptability of cancer cells to adverse environments. Herein, we will review some of the most important cancer-related AS events that play a functional role in the adaptation process set in motion by a tumour cell during both the early stages of development and progression of the pathology (Figure 2). Although cancer cells do not act “on purpose,” we present a figurative writing style to stress the dynamic nature of cancer cell adaptation.

### 2.1. Sustained Proliferative Signalling

A critical feature of tumorigenesis is uncontrolled cell proliferation, including the ability to grow in the absence of external stimuli. This skill is acquired through a myriad of abnormal modifications of growth factor signalling cascades and expression of their messengers and effectors. It is therefore not surprising that the powerful combinatorial effect conferred by AS is hijacked by cancer cells to increase the expression of isoforms whose activity promotes and sustains cell proliferation.

Homeostasis of growth control in cancer is often disrupted by constitutive activation of the RAS/MAPKs signalling pathway that plays a central role in cell proliferation, differentiation, and migration. Aberrant RAS activity may occur by several regulatory mechanisms that disrupt negative feedback loops or establish aberrant positive feedback loops in the pathway. The misregulation of AS of genes involved in the RAS pathway contributes to its activation in cancer, thus enhancing cell proliferation. An example is represented by the RAS-activated A-RAF kinase. AS of* A-RAF* is modulated by the splicing factor (SF) hnRNP A2, which represses the production of a short dominant-negative isoform in favour of the full-length transcript. Aberrant regulation of this splicing event leads to constitutive activation of the RAS/MAPKs pathway, cellular transformation, and increased proliferation [29]. Similarly, AS of* B-RAF* (V600E), a mutation present in 50% of metastatic melanomas, might result in being critical for this cancer and its treatment. A recent study involving experimental cell culture models and patient samples showed the existence of B-RAF (V600E) splice isoforms that lack the RAS binding domain and promote resistance to chemotherapy [30].

Hyperactivation of transmembrane receptors upstream of RAS can also contribute to favouring cancer-related AS events through positive feedback loops that modulate the activity of specific SFs. A typical example is represented by the epidermal growth factor receptor (EGFR), a tyrosine kinase receptor that plays a central role in cell proliferation and motility. EGFR pre-mRNA is alternatively spliced to generate a variant lacking exon 4 (EGFRΔ4). Skipping of this exon yields a receptor that is constitutively active and promotes proliferation. Notably, the EGFRΔ4 isoform is abundantly expressed in several cancers, such as glioma, prostate, and ovarian cancer [31]. Furthermore, a recent work documented that an active EGF signalling* per se* induces a massive reprogramming of AS. This effect was attributed to AKT-induced nuclear translocation of the SR protein kinase 1 (SRPK1). AKT binding to SRPK1 induces its autophosphorylation and dissociation from the HSP70 chaperone, which normally holds SRPK1 into the cytoplasm, thus favouring its nuclear translocation [32]. Once in the nucleus, SRPK1 can phosphorylate SR proteins and modulate the splicing pattern of several genes [32]. Since SRPK1 is usually localized in the cytoplasm in the absence of an extracellular signal and phosphorylates shuttling SR proteins in this cellular compartment [33, 34], stress signals might expand the effect of SRPK1 activation to the nucleus and influence also SR proteins that mostly reside in this compartment.

AS of the* CD44* gene also serves as a critical mechanism for a feed-forward loop that sustains activation of RAS/MAPK signalling [35]. CD44 is a transmembrane glycoprotein mediating the response of cells to their cellular microenvironment. CD44 is expressed in most tissues, where it functions in lymphocyte homing, adhesion, migration, and regulation of cell growth [36]. This variety of roles is favoured by the existence of multiple CD44 splice variants. The* CD44* gene is composed of 10 constitutively spliced exons and 10 variable exons, residing between constitutive exons 5 and 6. Upon mitogenic activation, the RAS/MAPK pathway positively regulates the activity of SAM68 and SRm160, two SFs that promote inclusion of variable exons in CD44 [37, 38]. The newly synthesized CD44v6 isoform, containing variable exon 6, forms complexes with receptor tyrosine kinases (RTKs) that promote RAS/MAPK activation and cell cycle progression [35].

Another oncogenic AS event that sustains uncontrolled proliferation of cancer cells affects the cyclin D1 (*CCND1*) protooncogene. Cyclin D1 associates with the cyclin-dependent kinase 4 (CDK4) to drive progression through the G1 phase of the cell cycle. Importantly, cyclin D1 expression is often deregulated in cancer cells [39, 40]. This gene encodes for two alternative transcripts: the common cyclin D1a isoform and the prooncogenic cyclin D1b isoform. In prostate epithelial cells, the canonical cyclin D1a isoform is involved in a negative feedback loop that controls proliferation. Cyclin D1a interacts with and represses the transcriptional activity of the androgen receptor (AR), which orchestrates the proliferation and activity of prostate cells [41]. By contrast, although the cyclin D1b isoform is capable of driving the G1/S transition of the cell cycle and to interact with AR, it does not repress its transcriptional activity, thereby interrupting this negative feedback [41]. Notably, two SFs that are often upregulated in cancer cells [42, 43], SRSF1 and SAM68, promote cyclin D1b splicing in prostate cancer cells [44, 45]. SAM68-dependent regulation of cyclin D1b splicing represents another clear example of how activated signalling pathways modulate cancer-related AS events by influencing the activity of specific SFs. Indeed, activation of the RAS/MAPKs pathway enhanced SAM68 binding affinity for cyclin D1 RNA and SAM68-dependent cyclin D1b splicing, whereas SAM68 phosphorylation by SRC-family kinases (SFKs) counteracted these activities [45]. Thus, these studies indicate how upregulation of two oncogenic SFs can unleash prostate cancer cells from the cyclin D1a/AR negative feedback that limits excessive proliferation of the epithelial cells in the normal organ.

### 2.2. Induction of Angiogenesis

Angiogenesis is the physiological process yielding new blood vessels. Neoangiogenesis normally occurs during embryogenesis and fetal development in response to the need for oxygen and nutrients of the growing mass of cells forming new tissues and organs. A similar situation occurs during tumorigenesis, when cancer cells begin to proliferate within a steady-state adult tissue. Growth of the tumour mass depletes the host tissue of nutrients and oxygen, causing starvation and promoting the formation of new vessels as an adaptive response. Tumour-associated neoangiogenesis provides cancers cells with access to blood circulation, thus facilitating tumour growth.

The main growth factors promoting angiogenesis are the vascular endothelial growth factors (VEGF) [46]. In humans, the VEGF family consists of five ligands and three signalling receptors. The ligands, VEGF-A-D and placental growth factor, are all alternatively spliced to yield isoforms with opposite or, in some cases, unknown function. Since* VEGF-A* AS is altered in a number of cancers, such as metastatic melanoma, neuroblastoma, and renal, prostate, colorectal, and bladder cancers [47], this gene is also the most studied of the family.

The alternative splice variants of* VEGF-A* exert different effects on tissue and tumour growth due to their opposing effects on angiogenesis. Several regulatory mechanisms that are critical for the splicing of this gene have now been identified. The best-known one consists in alternative usage of two 3′ ss in* VEGF-A* exon 8 [48]. SRSF1 and SRSF5 (SRp40) promote usage of the proximal 3′ ss, thus favouring the production of mRNAs encoding proangiogenic proteins [49]. By contrast, SRSF6 (SRp55) and SRSF2 (SC35) facilitate the selection of the distal 3′ ss, resulting in production of the antiangiogenic VEGFb isoform [49].

Signalling pathways evoked in cancer cells by the surrounding environment can mediate the balance between antiangiogenic and proangiogenic VEGF isoforms [50, 51]. Such regulation occurs either by direct control of their phosphorylation status by signalling kinases or by indirectly regulating splicing factor kinases involved in their posttranslational modifications. An example of indirect regulation is illustrated by insulin-like growth factor-1- (IGF-1-) mediated activation of protein kinase C (PKC) signalling, which in turn positively regulates SRPK1-dependent phosphorylation of SRSF1 and SRSF1-dependent* VEGF-A* AS [51]. A similar regulatory mechanism is also observed in prostate cancer, where selective upregulation of proangiogenic VEGF is under the direct control of SRPK1-regulated SRSF1 activity [50]. Importantly, genetic or pharmacological interference with SRPK1 activity caused a switch in the expression of proangiogenic towards antiangiogenic VEGF splice isoform, resulting in decreased microvessel density and reduced tumour growth [50]. Thus, the upregulation of SRPK1 and SRSF1 activity frequently observed in human cancers might contribute to the ability of the tumour mass to promote neoangiogenesis and redirect the blood stream towards itself. Alternatively, SRSF1-dependent* VEGF-A* AS may be indirectly regulated by the transcription factor WT1, encoded by the Wilms' tumour gene (*WT1*) [52]. It was shown that WT1 represses the transcription of* SRPK1* by directly binding to its promoter. This effect results in reduced SRPK1-dependent SRSF1 phosphorylation and inhibition of the production of prooncogenic VEGF isoform. Importantly, the authors demonstrated that in WT1 mutant cells* SRPK1* is highly expressed, SRSF1 is hyperphosphorylated, and VEGF prooncogenic isoforms are abundant, causing abnormal angiogenesis [52].

Recent evidence describes other novel regulatory circuits underlying the* VEGF-A* gene regulation that do not depend on the activity of SR proteins and/or on different usage of the 3′ ss in exon 8. For instance,* VEGF-A* AS is modulated by the alternatively spliced isoforms of the splicing factor T cell intracellular antigen (TIA-1). AS of* TIA-1* leads to expression of a truncated protein, called short TIA-1 (sTIA-1) in some cancer cells [53]. sTIA-1 competes with the binding of full-length TIA-1 to VEGF-A mRNA, thus favouring the production of the prooncogenic VEGF-A isoform, angiogenesis, and tumour growth in animal models. Notably, sTIA-1 expression positively correlates with advanced tumour stage in colorectal carcinoma (CRC) patients, supporting its prooncogenic function [53].

In addition to the well-studied antiangiogenic (VEGFb) and proangiogenic (VEGF)* VEGF-A* isoforms, a novel isoform named VEGF-A “extended” (VEGFAx), which displays antiangiogenic activity, was recently described [54]. In line with its inhibitory function on angiogenesis, VEGFAx expression levels are reduced in high-grade CRC tumours with respect to normal human colon mucosa. This isoform is produced by an uncommon regulatory mechanism, called programmed translational readthrough (PTR). This process is due to the presence of a* cis*-acting element that directs protein translation to continue beyond the canonical stop codon, with translation stopping at an alternative downstream stop codon. A recognition element for hnRNP A2/B1 was identified in the Ax region and loss of this recognition site, by either mutation of the sequence or knockdown of hnRNP A2/B1, reduced expression of VEGFAx [54].

AS of the* VEGF-A* gene may also be affected by epigenetic mechanisms. Chromatin features can directly affect splicing outcome by physically coupling the transcription machinery with the splicing apparatus via chromatin-binding adaptor proteins. The latter recognize exons or introns enriched in particular histone modifications and, in turn, recruit splicing regulators to nascent pre-mRNAs [10]. Using a high-throughput screen,* VEGF-A* was identified as a main target for chromatin-mediated AS regulation [55]. The authors showed that H3K9 methylation operated by the methyltransferase EHMT2 favours recruitment of the chromatin-binding protein HP1*γ* and its associated partner SRSF1 with the VEGF pre-mRNA, thus modulating its AS.

These examples illustrate the complexity of the regulation underlying VEGF-A pre-mRNA processing and translation and highlights how this process amplifies the escape routes available for cancer cells to adapt to an adverse environment.

### 2.3. Invasion and Metastasis

More than 90% of cancer-related deaths are due to metastasis and spread of cancer cells to multiple tissues and organs. The ability to form metastasis is probably the most complex task for cancer cells, which need to migrate from the primary tumour, intravasate, survive in blood, extravasate, and colonise different new environments. This implies an incredible phenotypic plasticity, which is largely due to a process called epithelial to mesenchymal transition (EMT) and the reverse, mesenchymal to epithelial transitions (MET) [56]. Through EMT, epithelial cells undergo an extensive reorganization of cytoskeletal architecture, with loss of intercellular junctions and cell polarity and acquisition of an elongated, fibroblast-like shape, thus acquiring invasive capabilities. EMT physiologically pertains to embryogenesis, when cells migrate to shape new organs, but it is adopted by cancer cells to generate metastases.

The ability of cancer cells to undergo EMT relies on the activation of a specific gene expression program in response to extracellular cues. Several interconnected regulatory networks drive EMT and modulation of any of them elicits profound effects on the others. The most extensively studied network is built around the transcription factors SNAIL, SLUG, ZEB1/2, and TWIST. Cues from the tumour microenvironment favour the expression of these factors and trigger a global change in gene expression that underlies EMT [57]. Nevertheless, other regulatory layers, including co- and posttranscriptional control by AS and small noncoding RNAs, interconnect with the transcriptional program and in some case can substitute or activate it, setting in motion critical aspects of EMT-associated phenotypic changes [57].

Many EMT-related genes generate AS variants encoding for proteins with essential functions in EMT and this topic has been recently reviewed elsewhere [57–59]. Herein, we wish to highlight few of the most relevant and well-described events. During EMT, several adhesion molecules specific of epithelial or mesenchymal cells are regulated through AS, such as CD44, p120-catenin (*CTNND1*), and MENA (*ENAH*) proteins [57]. For instance, AS of the* CD44* gene is tightly regulated during EMT in breast cancer cells.* CD44* AS is governed by the epithelial splicing regulatory protein 1 (ESRP1), a SF that stimulates inclusion of variable exons (CD44v isoforms). During EMT, ESRP1 levels drastically decrease, leading to the upregulation of the standard isoform (CD44s), which contributes to the formation of EMT-associated recurrent breast cancer in mice [60]. ESPR1 also positively regulates the production of the epithelial isoform MENA11a, which inhibits the migratory ability of breast cancer cells and is able to counteract the invasive activity of the mesenchymal MENAΔv6 isoform of the same gene [61].

The ESRP family members (ESRP1 and ESRP2) are so far the only known SFs exhibiting epithelial cell-type-specific expression and that undergo pronounced changes in expression during EMT [62, 63]. High-throughput experimental approaches revealed a high-affinity ESRP-binding motif (with UGG as a core motif) and a predictive “RNA map” that governs ESRP1/2 activity [64]. Importantly, downregulation of ESRP proteins during EMT affects splicing of a large number of these target genes, indicating that ESRPs are key players in this cancer-related cellular transition.

Other tissue-specific and more ubiquitously expressed SFs, such as the RBP FOX1 homologue (RBFOX), CUGBP Elav-like family (CELF), muscleblind-like protein (MBNL), SR proteins, and hnRNPs, also play a role in EMT [58]. For instance, hnRNP A1 has been recently implicated in the induction of the RAC1b isoform of the GTPase RAC1 [65], which is known to induce EMT through generation of reactive oxygen species (ROS) and induction of SNAIL expression [66]. hnRNP A1 negatively regulates RAC1b splicing by binding to RAC1 alternative exon 3b and inhibiting its inclusion; treatment with matrix metalloproteinase-3 (MMP-3) inhibits hnRNP A1 binding to exon 3b, thus relieving its repressive activity and favouring RAC1b splicing in mammary epithelial cells [65]. Conversely, in colorectal cells RAC1b splicing is positively regulated by the SR protein SRSF1 in a SRPK1-regulated manner [67]. Recent evidence shows indeed that the knockdown of SRPK1 or inhibition of its catalytic activity reduced phosphorylation and subsequent translocation of SRSF1 to the nucleus, limiting its availability to promote the inclusion of alternative exon 3b into the RAC1b pre-mRNA [67]. Thus, although a direct competition between SRSF1 and hnRNP A1 in RAC1b splicing regulation has not been demonstrated, it is tempting to speculate that epithelial or mesenchymal phenotype of a cancer cell could be modulated by the balance in the activity of these SFs and the consequent effect on RAC1b splicing.

SRSF1 also regulates the splicing of the tyrosine kinase receptor RON by inhibiting inclusion of exon 11 [68]. The resulting ΔRON isoform is unable to undergo proteolytic cleavage, rendering the protein constitutively active and conferring increased motility to cancer cells [68, 69]. Importantly, cancer cells modulate the expression levels of SRSF1, splicing of the ΔRON isoform, and induction of EMT in response to external cues from the surrounding environment. This process is orchestrated by a splicing cascade relying on phosphorylation/activation of the SAM68 by the extracellular regulated protein kinases (ERK1/2). Once activated, SAM68 promotes inclusion of a cryptic intron in the 3′ untranslated region of SRSF1 mRNA, thus inhibiting its degradation by nonsense mediated decay (NMD) [70].

Altogether, these observations indicate that AS plays a major role in EMT by establishing a specific program of splice variants of genes important for epithelial and mesenchymal cell morphology and motility. These observations raise the intriguing possibility that abnormal changes in splicing can steer cancer cells towards malignant progression through a partial EMT, without the need for canonical transcriptional reprogramming.

### 2.4. Resisting Cell Death

Apoptosis (also called programmed cell death) is a death process characterized by shrinkage of the cell and its nucleus. The apoptotic machinery is composed of both upstream regulators and downstream effector components. These players receive and integrate extracellular or intracellular cell death-inducing signals, giving rise to extrinsic and intrinsic apoptotic programs [26]. Both pathways culminate in a proteolytic cascade exerted by caspases. Once an insult hits a cell, it is the balance between pro- and antiapoptotic factors that determines cell fate.

Although apoptosis serves as a natural barrier to eliminate cells that develop aberrant features, transformed cells have developed a variety of strategies to limit or circumvent it. One of such strategies consists in modulation of AS to shift expression from pro- to antiapoptotic isoforms of several genes. Below, we will summarize some examples of apoptosis-related AS events that occur in the tumour microenvironment. As can be inferred from the list, each AS event is finely regulated by many SFs exhibiting synergistic or opposing functions. The cancer cell exploits the cooperation or competition between them to establish regulatory mechanisms that favour the production of the splicing isoform suitable for survival.

The death receptor* FAS* (an upstream regulator that receives extracellular death signals induced by the FAS ligand) and* CASP9* and* CASP8* (initial executioners of apoptosis) genes are regulated by AS, giving rise to splice isoforms with pro- or antiapoptotic roles. For instance, inclusion or skipping of FAS exon 6, respectively, generates two functionally distinct receptors, a membrane-bound protein with proapoptotic function and a soluble form with antiapoptotic function [71, 72]. TIA-1, TIAR-1 (TIA-1 related) [73], and EWS (Ewing sarcoma protein) [74] positively regulate* FAS* splicing by favouring the assembly of the spliceosome on the 5′ and 3′ ss of exon 6, resulting in the generation of the proapoptotic isoform. By contrast, PTB/hnRNP I [73], RBM5 [75], HuR [76], and hnRNP C1/C2 [77] negatively regulate exon 6 splicing in favour of the antiapoptotic FAS isoform.

Caspase-9 is the most studied family member in terms of AS. The* CASP9* gene generates two splice variants, the proapoptotic caspase-9a and the antiapoptotic caspase-9b, which differ for the inclusion or exclusion of a four-exon cassette (exons 3, 4, 5, and 6), respectively [78, 79]. SRSF1 promotes the inclusion of the exon cassette contributing to the generation of caspase-9a proapoptotic isoform in non-small cell lung cancer (NSCLC) cells [80]. However, constitutive activation of the PI3K/AKT pathway in these cells repressed this activity [80]. On the other hand, hnRNP L promotes skipping of the exon cassette to generate the antiapoptotic caspase-9b protein [81]. Interestingly, the expression level and the phosphorylation status of hnRNP L strongly influence the outcome of this AS event. Overexpression of hnRNP L in NSCLC cells, but not in nontransformed cells, lowers the caspase-9a/9b ratio, favouring the oncogenic isoform. The physiological relevance of this mechanism was confirmed by the complete loss of tumorigenic capacity in a mouse xenograft model of NSCLC cells depleted of hnRNP L [81].

The cancer-restricted role of hnRNP L in caspase-9 AS is apparently due to NSCLC-specific phosphorylation of hnRNP L on Ser52, suggesting that cancer cell developed a device to switch an ubiquitous RBP into a prooncogenic protein through a specific posttranslational modification.

Many other apoptosis-related genes are also subjected to AS regulation. The* BCL-X* (*BCL2L1*) gene contains 3 exons and encodes two splice variants [82]. Two alternative 5′ ss are present in exon 2: selection of the canonical one at the end of the exon yields the long, antiapoptotic variant BCL-X_L_, whereas selection of the distal one located upstream in the exon produces the short, proapoptotic variant BCL-X_S_ [82]. Several SFs have been shown to modulate* BCL-X* splicing. HnRNP H, F, and I (PTB) [83, 84], SAM68 [85], the RBPs RBM25 [86], and RBM11 [87] were all shown to promote splicing of the proapoptotic BCL-X_S_ variant. By contrast, the SFs SAP155 [88], SRSF9 [89], hnRNP K [90], and SRSF1 [85, 91] enhance splicing of the antiapoptotic BCL-X_L_. The balance of BCL-X isoforms is affected in a large number of cancer cell lines and human cancer samples, and fine-tuned regulation of this AS event can determine the cell fate in response to various stresses [85, 92, 93].

These examples highlight how different families of SFs are employed by cancer cells to coordinate splicing regulation and to promote cell survival in response to the hazards imposed by the variable environmental conditions, gaining an advantage with respect to nontransformed cells.

### 2.5. Deregulating Cellular Energies

The uncontrolled cell proliferation that characterizes cancer cells involves adjustments of energy metabolism in order to favour a rapid growth and division of tumour cells even in adverse microenvironments. Under aerobic conditions, cells produce energy via glycolysis in the cytosol (this reaction allows the conversion of glucose to pyruvate) and then via oxidative phosphorylation in the mitochondria (this reaction allows the conversion of pyruvate to carbon dioxide). Under anaerobic conditions, glycolysis is favoured compared to oxygen-consuming mitochondrial oxidative phosphorylation. Cancer cells, however, primarily use glycolysis, by reprogramming their glucose metabolism and energy production regardless of oxygen supply. This cancer-related process, called “aerobic glycolysis,” was already discovered in 1930 by Warburg [94, 95]. The efficiency of ATP production insured by glycolysis is lower than that provided by mitochondrial oxidative phosphorylation. However, an increased glycolysis provides advantages to cancer cells by allowing a more efficient utilization of glycolytic intermediates in other biosynthetic pathways that favour proliferation also in presence of limited amounts of nutrients [96]. This reliance on glycolysis can be further accentuated under the hypoxic conditions occurring within the growing tumour mass.

AS of key metabolic enzymes partially governs the metabolic switch that characterizes cancer cell metabolism. A well-studied example is that of pyruvate kinase (PKM), an enzyme that catalyses the conversion of phosphoenolpyruvate (PEP) to pyruvate [97]. The* PKM* gene encodes two alternative splice variants through usage of mutually exclusive exons [97]. The PKM1 isoform, produced when exon 9 is included in the mature transcript, is normally expressed in adult life and stimulates mitochondrial oxidative phosphorylation. PKM2, generated by inclusion of exon 10, is exclusively expressed during embryonic development and promotes aerobic glycolysis. However, PKM2 is typically reexpressed in cancer cells where it confers oncogenic features [97–99]. Indeed, replacement of PKM2 with PKM1 in lung tumour cells correlated with impaired tumour occurrence in mouse xenografts [97].* PKM* splicing in cancer cells is modulated by hnRNP A1, hnRNP A2, and hnRNP I/PTB, which cooperate to promote splicing of PKM2 by binding to sequences flanking exon 9 and repressing its inclusion [100, 101]. Notably, all three hnRNPs are overexpressed in several cancers [23, 24] and their expression can be coordinated by the oncogenic transcription factor MYC [101]. Thus, during neoplastic transformation upregulation of MYC activity and of these SFs might predispose the cell to alter its energy metabolism through modulation of* PKM* AS. This transition would render the cancer cell less susceptible to starvation and/or other unfavourable metabolic conditions occurring in the tumour microenvironment.

### 2.6. Chemotherapy Resistance

**Figure 3:**
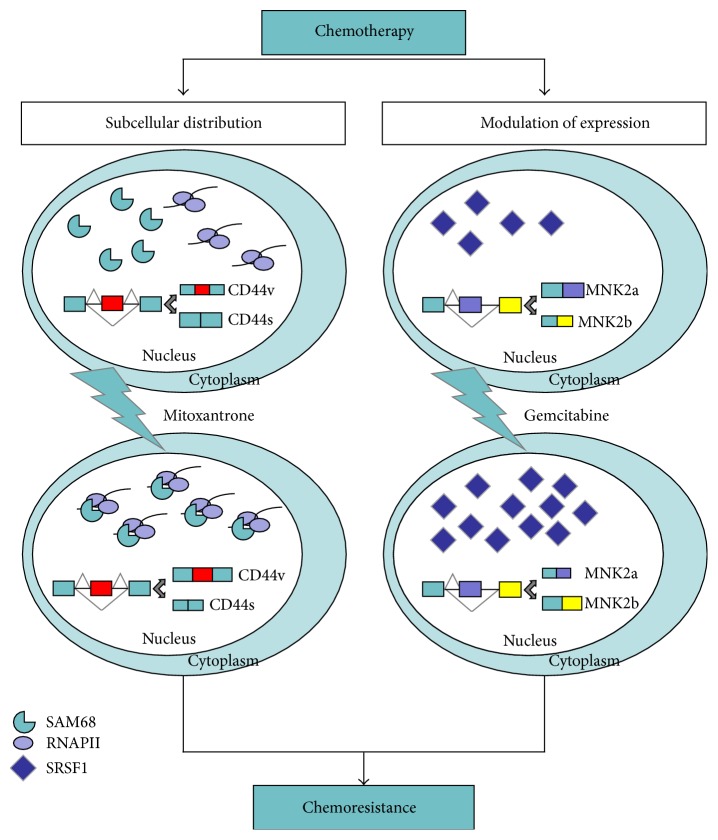
Regulation of chemoresistance via alternative messenger RNA splicing. Chemotherapy can affect the subnuclear distribution of SAM68 (left panel) and the expression of SRSF1 (right panel), thus modulating AS of cancer-related variants (CD44v and MNK2b, resp.) and contributing to chemoresistance. Green boxes indicate constitutive exons; red, yellow, and violet boxes indicate variable exons.

Surgery, radiation, and chemotherapeutic drugs are the standard approaches for cancer treatment. Radiation and chemotherapy mainly act by inducing cancer cell death. Although most tumours respond to chemotherapy at first, some cancer cells often survive treatments, expand, and acquire chemoresistance causing disease relapse. The mechanisms by which cancer cells adapt or are selected for their resistance to treatments vary with cancer type and from patient to patient. Most of these mechanisms causing chemotherapy resistance have been elegantly described elsewhere and mainly involve mutations and/or altered expression of genes and proteins [102]. Among these, AS participates in the process of acquired chemoresistance by controlling the expression of cancer-related splice variants that contribute to cancerous phenotype (Figure 3). Herein, we illustrate some examples of how AS allows cancer cells to adapt to tumour microenvironment, under conditions where normal cells would undergo cell death, and to overcome the chemotherapy-mediated selective pressures.

An interesting example of AS adaptive response driven by chemotherapy is provided by the switch from cyclin D1a to cyclin D1b in breast cancer cells. Upon treatment of MCF-7 cells with cisplatin and the estrogen receptor antagonists 4-hydroxy tamoxifen and ICI 182780, endogenous protein cyclin D1a expression is strongly reduced, whereas oncogenic cyclin D1b splice variant is maintained and confers chemoresistance [103].

The HER2-targeted therapy using trastuzumab is widely used for the treatment of patients with metastatic breast tumours overexpressing HER2, a member of EGFR family of receptor tyrosine kinases. Although the search for a somatic* HER2* oncogenic mutation in* HER2*-amplified breast tumours has failed to identify a promising activating genetic lesion [104–106], the existence of HER2 isoforms that may influence trastuzumab response in breast tumours evidenced the key role of AS in chemoresistance [107–109]. A new* HER2* splice variant (HER2Δ16) with potent transforming activity was detected in several* HER2*-overexpressing breast cancer cell lines [108, 109] and primary tumours [107, 109]. Furthermore, the expression of HER2Δ16 is a tumour-specific molecular event and the vast majority of women with expression of HER2Δ16 develop locally disseminated node-positive breast cancer. Furthermore, tumour cell lines expressing HER2Δ16 are resistant to the HER2-targeted therapy trastuzumab [110]. The critical effector of HER2Δ16 tumorigenic properties is represented by SRC kinase. In fact, SRC kinase appears to function as a “master regulator” stabilizing HER2Δ16 protein expression and coupling HER2Δ16 to multiple mitogenic and cell motility pathways [110]. Cotargeting of HER2Δ16 and SRC kinase with the single agent tyrosine kinase inhibitor dasatinib resulted in SRC inactivation, destabilization of HER2Δ16, and suppressed tumorigenicity [110]. An important issue will be to characterize the cancer-specific splicing event leading to HER2Δ16 expression in breast cells. Understanding these mechanisms might indeed offer therapeutic perspective to counteract the activity of this oncogenic splice variant in breast cancers with poor prognosis.

Another SF involved in drug resistance is SPF45, a 45 kDa nuclear protein [111, 112]. SPF45 is highly expressed in numerous carcinomas including bladder, breast, colon, lung, ovarian, pancreatic, and prostate. Forced overexpression of SPF45 in HeLa cells demonstrated a 4–7-fold increase in resistance to doxorubicin. Ectopic SPF45 expression in the A2780 ovarian cancer cells induced a multidrug resistant phenotype, inducing 3–21-fold resistance to a variety of chemotherapeutics with differing mechanisms of action, including carboplatin, vinorelbine, doxorubicin, etoposide, mitoxantrone, and vincristine [111]. The mechanism underlying the multidrug resistant phenotype acquired upon SPF45 overexpression is still unknown but probably relies on misregulation of AS of its targets [113, 114]. Few splicing targets of SPF45 are currently known. SPF45 promotes the proapoptotic transmembrane receptor FAS pre-mRNA [114], but this activity is repressed by both mitogenic (ERK1/2) and stress-response (p38 and JUN N-terminal kinases) MAPK-dependent phosphorylation in cancer cells [113]. SPF45 is also phosphorylated by CLK1 on multiple serine residues and this posttranslational modification regulates alternative ss utilization by SPF45 and its intranuclear localization [115]. Furthermore, stable SPF45 overexpression in SKOV-3 cells induces enhancement of fibronectin 1 expression and regulates fibronectin 1 AS by enhancing inclusion of the EDA region into fibronectin transcripts [113]. Since inclusion of EDA region in fibronectin enhances the migratory capacity of embryonic cells and tissue, SPF45 overexpression might contribute to promote metastasis* in vivo* by modulating this AS event. Thus, full elucidation of the spectrum of AS events regulated by this SF in cancer cells might reveal pathways involved not only in acquisition of chemoresistance but also in other key oncogenic features.

Although a large spectrum of AS events associated with chemoresistance has been described [116–118], much less is known about the mechanisms activated during chemotherapy that result in the observed splicing changes. In this regard, genotoxic stress may cause a subcellular redistribution of many RBPs and/or modify their activity through posttranslational modifications as an attempt of the cancer cell to adapt to the hostile environment [119–121]. The subcellular localization of SAM68 and other RBPs was affected by treatment of prostate cancer cells with mitoxantrone (MTX), a topoisomerase II inhibitor used in chemotherapy, partially altering the cellular AS pattern [119]. Another regulatory mechanism through which tumour cells acquire resistance involves the modulation of expression of specific SFs or of cancer-related splicing variants and/or of their counterparts. Treatment of pancreatic ductal adenocarcinoma (PDAC) cells with gemcitabine induced the upregulation of SRSF1 that, in turn, regulates AS of mitogen activated protein kinase (MAPK) interacting kinase 2 (*MNK2*) in favour of MNK2b isoform [122]. SRSF1-dependent AS of MNK2b following gemcitabine treatment conferred increased resistance of PDAC cells to chemotherapeutic drug, identifying a novel chemotherapy-mediated adaptation response through AS in PDAC cells [122]. Notably, a recent report showed that MNK2a behaves as tumour suppressor in breast cancer, whereas the alternative MNK2b splice variant was prooncogenic [123]. Thus, it appears that upregulation of SRSF1 in response to genotoxic stress confers resistance to treatments by switching this splicing event in favour of the prooncogenic MNK2b variant.

These observations suggest that AS changes induced by chemotherapeutic treatment represent an important side-effect, which may contribute to therapy resistance. These aspects need to be taken into account for the development of new therapeutic protocols that could exploit the combined usage of canonical chemotherapy with novel pharmaceutical tools targeting the adaptive splicing response associated with treatments.

## 3. Concluding Remarks

As shown by the several examples illustrated in this review, AS plays a key role in the rearrangement of gene expression, thus enabling cancer cells to adapt to the adverse conditions encountered during the transformation process and to evade different therapeutic approaches. At the same time, these observations suggest that the splice variants aberrantly expressed by cancer cells might represent suitable targets for the development of new antitumor therapies, in particular those whose prognostic or diagnostic values have already been demonstrated [124]. Redirecting aberrant splicing events or inhibiting the activity of oncogenic splice variants can represent a valuable approach to increase cancer cells sensitivity to canonical chemotherapies, which could be exploited in new combined therapies. As an example, susceptibility of NSCLC cells to different chemotherapeutic drugs can be enhanced by RNA interference of the expression of the antiapoptotic splice variant caspase-9b of the* CASP9* gene [125]. Notably, one of the advantages of therapeutically targeting alternative splice variants is the possibility to act on two different fronts: on one hand, therapies targeting the specific activity of the oncogenic splice variant could be developed; on the other hand, the mechanisms driving the aberrant splicing event could also be targeted. In light of this, great interest has arisen for studies exploiting antisense oligonucleotide (ASO) to redirect splicing of tumoral variants towards a nontumoral isoform. This approach has been recently shown to be possible for the PKM2/PKM1 [126] and the BCL-X_L/S_ [93] splicing switch. The high therapeutic value of the ASO approach is strongly supported by recent studies demonstrating the good bioavailability and efficacy of an ASO redirecting* SMN2* splicing for the treatment of SMA animal models [127].

Thus, it is certainly possible to envision the development in the near future of new personal anticancer therapies targeting the specific splicing-alterations of each patient, whose identification will be ensured by the novel and rapidly evolving high-throughput sequencing techniques that allow genome-wide profiling of cellular transcriptomes, even at a single cell-resolution [21].
